# Pharmacokinetics and Pharmacodynamics of a Nanostructured Lipid Carrier Co-Encapsulating Artemether and miRNA for Mitigating Cerebral Malaria

**DOI:** 10.3390/ph17040466

**Published:** 2024-04-06

**Authors:** Veera Venkata Nishanth Goli, Spandana Tatineni, Umme Hani, Mohammed Ghazwani, Sirajunisa Talath, Sathvik Belagodu Sridhar, Yahya Alhamhoom, Farhat Fatima, Riyaz Ali M. Osmani, Umamaheshwari Shivaswamy, Vichitra Chandrasekaran, Bannimath Gurupadayya

**Affiliations:** 1 Department of Pharmaceutical Chemistry, JSS College of Pharmacy, JSS Academy of Higher Education and Research, Shivarathreeshwara Nagara, Mysuru 570015, India; nishanthgoli81@gmail.com (V.V.N.G.); spandanatatineni4@gmail.com (S.T.); 2 Department of Pharmaceutics, College of Pharmacy, King Khalid University, Abha 62529, Saudi Arabia; uahmed@kku.edu.sa (U.H.); myghazwani@kku.edu.sa (M.G.); ysalhamhoom@kku.edu.sa (Y.A.); 3 Department of Pharmaceutical Chemistry, RAK College of Pharmacy, RAK Medical and Health Sciences University, Ras Al Khaimah 11172, United Arab Emirates; sirajunisa@rakmhsu.ac.ae; 4 Department of Clinical Pharmacy & Pharmacology, RAK College of Pharmaceutical Sciences, RAK Medical and Health Sciences University, Ras Al Khaimah 11172, United Arab Emirates; sathvik@rakmhsu.ac.ae; 5 Department of Pharmaceutics, College of Pharmacy, Prince Sattam Bin Abdulaziz University, Al-Kharj 11942, Saudi Arabia; f.soherwardi@psau.edu.sa; 6 Department of Pharmaceutics, JSS College of Pharmacy, JSS Academy of Higher Education and Research, Shivarathreeshwara Nagara, Mysuru 570015, India; riyazosmani@gmail.com; 7 Department of Microbiology, JSS Academy of Higher Education and Research, Mysuru 570015, India; umamaheshwari@jssuni.edu.in; 8 Department of Pharmacology, JSS College of Pharmacy, JSS Academy of Higher Education and Research, Shivarathreeshwara Nagara, Mysuru 570015, India; cvichy97@gmail.com

**Keywords:** nanostructured lipid carrier, miRNA, pharmacokinetics, intranasal delivery, brain targeting, NOX2, IL-6, cerebral malaria

## Abstract

Cerebral malaria (CM), a severe neurological pathology caused by *Plasmodium falciparum* infection, poses a significant global health threat and has a high mortality rate. Conventional therapeutics cannot cross the blood–brain barrier (BBB) efficiently. Therefore, finding effective treatments remains challenging. The novelty of the treatment proposed in this study lies in the feasibility of intranasal (IN) delivery of the nanostructured lipid carrier system (NLC) combining microRNA (miRNA) and artemether (ARM) to enhance bioavailability and brain targeting. The rational use of NLCs and RNA-targeted therapeutics could revolutionize the treatment strategies for CM management. This study can potentially address the challenges in treating CM, allowing drugs to pass through the BBB. The NLC formulation was developed by a hot-melt homogenization process utilizing 3% (*w*/*w*) precirol and 1.5% (*w*/*v*) labrasol, resulting in particles with a size of 94.39 nm. This indicates an effective delivery to the brain via IN administration. The results further suggest the effective intracellular delivery of encapsulated miRNAs in the NLCs. Investigations with an experimental cerebral malaria mouse model showed a reduction in parasitaemia, preservation of BBB integrity, and reduced cerebral haemorrhages with the ARM+ miRNA-NLC treatment. Additionally, molecular discoveries revealed that nicotinamide adenine dinucleotide phosphate oxidase 2 (NOX2) and Interleukin-6 (IL-6) levels were reduced in the treated groups in comparison to the CM group. These results support the use of nanocarriers for IN administration, offering a viable method for mitigating CM through the increased bioavailability of therapeutics. Our findings have far-reaching implications for future research and personalized therapy.

## 1. Introduction

Cerebral malaria is a deadly neurological condition triggered by the insidious *Plasmodium falciparum*. It has a significant incidence and affects children who live in sub-Saharan Africa. Individuals who have endured such infections may be more vulnerable to long-term neurological impairments [1]. The invasion of parasites increases oxidative stress, which disturbs the delicate balance of redox reactions and results in abnormally elevated concentrations of reactive oxygen and nitrogen species (ROS/RNS) [2]. This oxidative and nitrosative stress surge can harm the host’s natural antioxidant defences. It is worth noting that astrocytes, neurons, and microglia exhibit complex immune responses in the presence of *P. falciparum*. The complex relationship between the parasite and the host’s neural components highlights the intricate pathophysiology of CM, providing insights into possible therapeutic interventions and preventive strategies [3]. In experimental cerebral malaria (ECM), the activation of astrocytes and microglial cells is triggered by proteins from both the parasite and the host. This activation releases unique signalling molecules, such as IL-6, cytokines, chemokines, and ROS. Elevated oxidative stress plays a crucial role in triggering tissue damage in the brain. This sequence of events unfolds as the dysfunction of endothelial cells occurs, resulting in the compromise integrity of the BBB. As a result, the compromised barrier integrity paves the way for neuroinflammation, marked by the release of molecules that induce inflammation [4,5]. This complex sequence of reactions ultimately leads to disruptions in brain function, emphasizing the interconnectedness of oxidative stress, vascular integrity, and neuroinflammation in the progression of ECM [6]. ROS contribute to the maintenance of various biological processes in the body, such as immune responses, cell growth, and cellular differentiation [7]. Multiple research investigations have produced valuable insights into the importance of nicotinamide adenine dinucleotide phosphate (NADPH) oxidase and NOX in the numerous physiological processes occurring in the brain. Numerous studies have demonstrated a connection between NOX expression and oxidative damage-related disorders, including Parkinson’s disease (associated with motor dysfunction) and Alzheimer’s disease (linked to cognitive impairment), which are associated with the generation of superoxides [8]. This generation of free radicals plays a significant role in increasing oxidative stress and causing hippocampal lesions in CM [9,10]. It is noteworthy that the suppression of NOX expression has shown great promise in addressing an array of central nervous system (CNS) disorders [11,12].

MicroRNAs (miRNAs) play a vital part in a broad spectrum of cellular processes, such as differentiation, cell proliferation, and programmed cell death. A notable factor is miR-223, which is generated by haematopoietic cells [13]. It significantly impacts the regulation of monocyte–macrophage differentiation, neutrophil recruitment, and proinflammatory responses [14,15]. During malaria infections, miRNA-223 exerts its effects through complex molecular mechanisms involving the inhibition of ICAM-1 through the phosphorylation of p38 MAP kinase (mitogen-activated protein kinase), JNK (c-Jun N-terminal kinase), and ERK (extracellular signal-regulated kinase) and further dysregulates the nuclear translocation of NF-kB p65 (Nuclear Factor-kappa B), which could have direct or indirect effects on parasite growth inhibition [16,17]. According to the research conducted by LaMonte et al., it has been predicted that miRNAs can impact gene expression in *Plasmodium falciparum* through a distinct mechanism. Specifically, these miRNAs can infiltrate *Plasmodium falciparum* cells and attach themselves to mRNAs, impeding their translation. Hence, the rise in miR-223 expression in mice suffering from CM suggests a potential immune reaction against parasites, warranting further investigation into the therapeutic possibilities of targeting miR-223 [18]. ARM, a semisynthetic derivative of artemisinin, has become increasingly crucial in innovative antimalarial therapeutic strategies endorsed by the World Health Organization (WHO) [19]. These approaches, which use several artemisinin compounds, have enhanced safety and tolerability. The molecular mechanism of ARM in reducing CM involves a multifaceted approach, including the inhibition of parasite growth by damaging the DNA and increasing ROS production, which further causes membrane depolarization. ARM also disrupts parasite sequestration by activating the heme cascade and the formation of c-radicals causing alkylation of the parasite proteins and induction of autophagy and apoptosis [20,21]. These mechanisms collectively contribute to the efficacy of artemether in alleviating symptoms, improving clinical outcomes, and reducing the mortality rates of CM. Recent investigations have revealed ARM’s powerful cytotoxic and anti-inflammatory properties, broadening its potential use in treating various diseases such as cancer, viral and fungal infections, sepsis, haemorrhage, and arthritis [22].

In the context of cerebral malaria, ARM has been shown to possess anti-inflammatory properties, which may contribute to its ability to reduce the expression of interleukin-6 (IL-6). IL-6 is a pro-inflammatory cytokine that plays a key role in orchestrating the immune response to infection. Studies have suggested that ARM can modulate the production of various cytokines, including IL-6, by suppressing inflammatory signalling pathways such as the NF-κB and MAPK pathways [23]. Additionally, ARM may inhibit the activation of immune cells, such as microglia and macrophages, which are major sources of IL-6 production in cerebral malaria [24]. Therefore, it is anticipated that ARM alone could attenuate IL-6 expression, potentially contributing to its therapeutic efficacy in mitigating neuroinflammation and improving clinical outcomes in CM patients. ARM is well-known for its hydrophobic nature; however, it cannot cross the BBB on its own and maintain a high concentration in cerebral tissues; thus, to address these challenges, diverse formulation strategies have been tested to make it a promising contender for treating cerebral disorders [25]. Although this drug shows potential, its limited brain vascular bioavailability is a problem that has prompted researchers to investigate other formulation options. Our research aimed to explore formulation strategies that work in concert with miRNA to boost ARM’s effectiveness.

Typically, individuals suffering from CM are given oral drugs, which, regrettably, have lower-than-ideal bioavailability and limited therapeutic benefits. The oral route poses certain obstacles, such as vulnerability to first-pass metabolism, enzyme degradation, swift clearance, and selective accumulation in peripheral tissues, all hindering the desired therapeutic outcomes [26]. Contemporary advancements in drug delivery technologies have induced researchers to investigate the possibilities of IN administration as a viable alternative. This approach provides benefits by potentially bypassing the BBB and enhancing the availability of therapeutic agents without the need for invasive procedures. The IN administration method allows for the direct transportation of drug molecules to the CNS, resulting in increased concentrations in the CNS. This effectiveness is backed by extensive pre-clinical evidence, demonstrating the potential of the IN route to utilize trigeminal, glymphatic, and olfactory pathways to achieve higher drug concentrations in the CNS. Numerous studies have demonstrated the efficacy of IN delivery, highlighting its capacity to target the CNS more precisely and offer improved therapeutic benefits over traditional delivery modalities [27].

Recently, nanocarriers have become a highly intriguing strategy for drug delivery, offering significant benefits compared to traditional techniques. Various carriers, including exosomes, nanoparticles of polymers, extracellular vesicles, and liposomes, can encapsulate and deliver drugs with precision to targeted tissues or cells. This precise delivery method minimizes any unintended effects and decreases the potential harm. In the context of brain inflammatory disorders, the application of nanocarriers has demonstrated great potential in administering drugs that focus on the underlying neurological processes associated with such conditions [28]. A recent study delved into the various strategies for enhancing the effectiveness and accuracy of nanocarriers to achieve better therapeutic outcomes [29]. Nanocarriers are an enticing field of study that has the potential to revolutionize drug delivery by improving drug effectiveness and safety across an extensive spectrum of ailments. In treating neurodegenerative illnesses and brain disorders, more effective and tailored nanocarriers have great potential [30]. Enhancements in emerging methods for drug delivery allow pharmaceutical researchers to leverage the intriguing possibilities of nanocarrier systems, resulting in enhanced clinical outcomes. These advancements have sparked interest in exploring IN delivery using carrier systems to encapsulate therapeutic agents [31]. However, enhancing benefits requires establishing appropriate release kinetics, optimizing dosages, and ensuring that medication molecules are active when they reach the absorption site. Employing biocompatible polymers with amphiphilic properties in nanocarrier systems offers significant benefits for transferring medicinal compounds. Because of their remarkable adaptability, these nanocarriers can be tailored to meet the objectives of a given research project and facilitate efficient dispersion [24,32]. A comprehensive investigation has been carried out on distinct kinds of NLCs to explore their potential to cross the BBB. Research communities are likely to be interested in this formulation strategy, which might lead to more investigations and evaluations of its appropriateness for IN delivery to treat numerous neurological disorders [33].

Our findings indicate that using NLCs loaded with miRNA and ARM can improve CM treatment. This method aims to enhance the system’s drug bioavailability and brain-targeting capabilities, potentially leading to more effective disease management. This investigation aimed to assess and examine the pharmacokinetic specifications, efficacy, and reliability of recently formulated NLCs in CM management. The outcomes of this research might offer a foundational basis for future investigations on the effective administration of medicinal compounds through the nasal route to treat disorders related to CM. NLCs offer a highly effective method of administering drugs to the desired site of action by efficiently crossing the BBB through the IN route. Including therapeutic agents in nanocarriers is crucial for ensuring the therapeutics’ secure and economical delivery to manage CNS disorders. Considering the scarcity of research on the successful administration of miRNAs through the nasal route, this study’s outcome provides significant knowledge and establishes an essential basis for future studies. The findings indicate the potential for the non-invasive IN administration of therapeutic compounds to the CNS, thus tackling the difficulties linked to treating neurodegenerative conditions.

## 2. Results

### 2.1. Formulation and Characterization of ARM+ miRNA-NLCs

**Figure 1:**
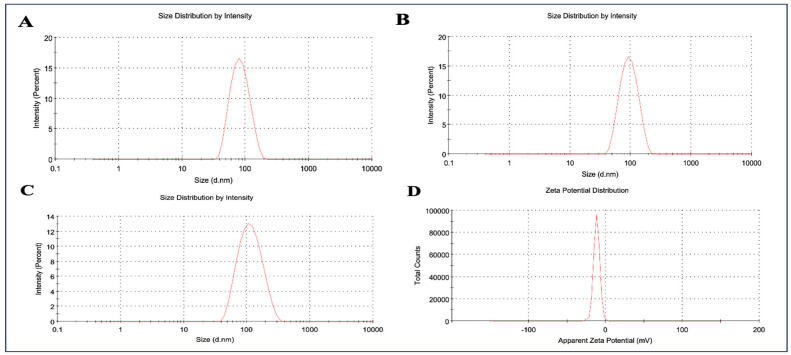
The dynamic light scattering data of the nanostructured lipid carrier (NLC) system showcasing (**A**) blank NLCs’ particle size of 76.84 nm; (**B**) ARM-NLCs’ particle size of 94.39 nm; (**C**) ARM+ miRNA-NLCs’ particle size of 94.39 nm; and (**D**) zeta potential of −11.8 mV for ARM+ miRNA-NLCs.

The particle size analysis of the prepared NLCs was performed by adopting the dynamic light scattering technique. The particle size of blank NLCs and ARM-NLCs was found to be 76.84 nm and 87.26 nm with a polydispersity index (PDI) of 0.107 and 0.107, respectively (Figure 1A,B). On the other hand, the particle size of ARM+ miRNA-NLCs was found to be 94.39 nm with a PDI of 0.122 (Figure 1C). As anticipated, the outcomes of the particle size analysis reflected an increase in particle size with the loading of ARM and miRNA without any aggregation or polydispersity of the particles. The zeta potential recorded for ARM+ miRNA-NLCs was −11.8 mV, as shown in Figure 1D. Higher entrapment levels were observed specifically at 93.06 ± 3.43%.

### 2.2. Evaluation of Linearity of Plasma Samples by Employing RP-HPLC

**Figure 2:**
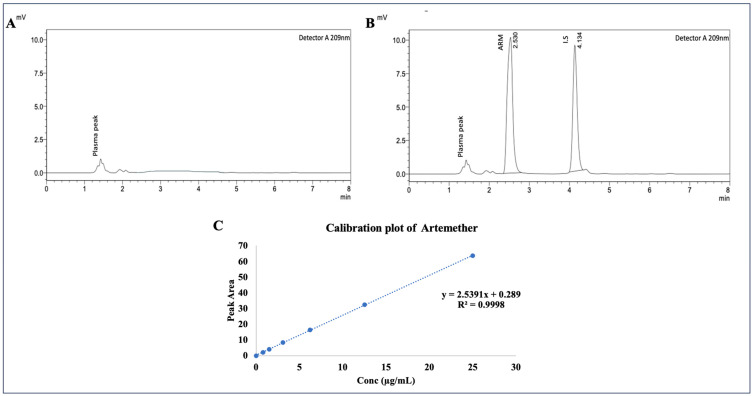
HPLC chromatograms for (**A**) blank plasma and (**B**) ARM, obtained at RT 2.5 and I.S. at RT 4.1 min, respectively, and (**C**) calibration plot for ARM.

To ensure the absence of endogenous plasma constituents, blank plasma samples were analysed initially. After that, rat plasma was isolated and spiked with standard solutions containing 10–1000 µg/mL ARM and 10 µg/mL I.S. (DHA). A calibration curve was generated for the linear range of 0.78–25 µg/mL, and Figure 2A,B depicts the blank plasma and ARM standard bioanalytical chromatograms. The calibration curves were plotted by graphing the ratio of ARM’s peak area to the internal standard. Using the least-squares approach, the mean linear regression equation for ARM in the concentration range of 0.78–25 µg/mL was calculated, yielding y = 2.5391x + 0.289, as portrayed in Figure 2C (the equation represents the concentration of ARM in plasma as x, and the peak area ratio of ARM to the I.S. as y). The ARM levels in the plasma were determined using the ratios of the ARM peak areas to I.S. peak areas. The calibration curve demonstrated remarkable linearity over the standard range under investigation during validation, with an R2 correlation value of 0.9998 for ARM. The limit of quantification (LOQ) was found to be 1.5 µg /mL at a predefined signal-to-noise ratio of about 10. The recovery was calculated at three distinct concentrations (1.5, 6.2, and 25 µg/mL ARM in plasma). The recovery range for ARM’s mean percentage was 91.42% to 97.29%. At 10 µg/mL, the mean percentage recovery for the internal standard was 97.17%. The lower limit of quantification (LLOQ) was within 20%, and the non-zero calibrators were within 15% of the nominal values, as per US FDA guidelines [34].

### 2.3. Agarose Gel Electrophoresis

**Figure 3:**
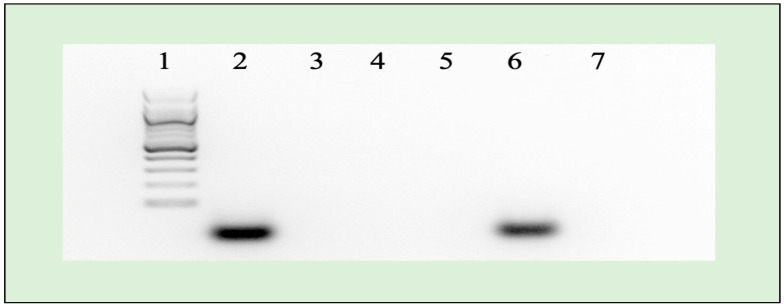
Agarose gel electrophoresis demonstrating complexation and stability of NLCs-miRNA system. Lane 1: marker; Lane 2: miRNA 223; Lane 3: NLC blank; Lane 4: NLC-ARM; Lane 5: NLC-ARM-miRNA; Lane 6: NLC-ARM-RNA-heparin; Lane 7: heparin.

The results confirmed the successful loading of miR-223 (10 nM final concentration) into the NLCs; there was a significant inhibitory effect on their migration when exposed to the applied electrical field. Interestingly, the utilization of heparin to release loaded miR-223 from the NLCs led to a migration pattern in the agarose gel that closely resembled the control conditions. This observation demonstrates that the loaded miR-223 remained intact throughout the release process, as displayed in Figure 3. The migration patterns observed in both the released miRNA and control group highlight the remarkable stability and protection provided by the NLCs.

### 2.4. Cellular Absorption Evaluation

**Figure 4:**
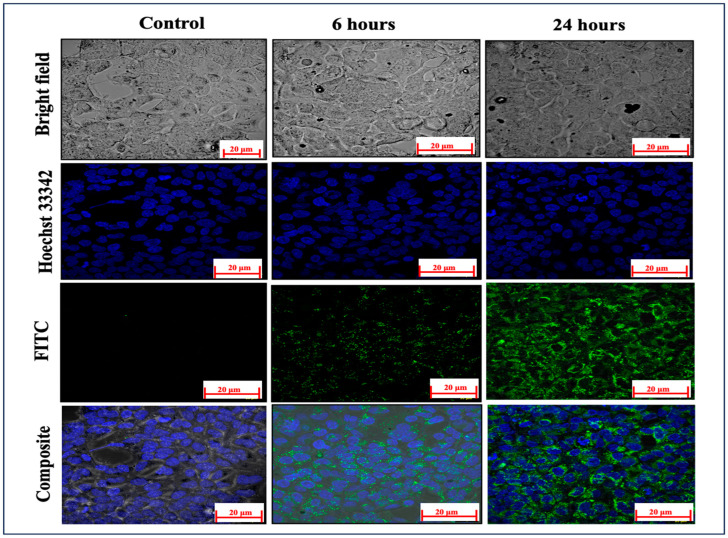
Results of confocal microscopy analysis, specifically focusing on the FITC signal intensity in NLCs after 6 and 24 h of incubation, blue represents Hoechst-stained cell nuclei and green represents FITC stained NLCs.

Evaluating the effective cellular absorption of medicines from their delivery vehicles is critical for establishing therapeutic efficacy. For the investigation of ARM-miRNA-NLCs, they were labelled with FITC dye, a prominent dye used for fluorescence contrast imaging. The FITC dye was chemically conjugated via covalent bonding with the NLC lipid. Detailed data of the FT-IR spectral analysis confirming the chemical conjugation of FITC with the NLCs is provided in the ‘Supplementary Materials’. The capacity of the ARM-miRNA-NLCs to infiltrate the cell membranes and penetrate into the cytoplasm is critical in determining the influence on HEK cells; after 6 and 24 h, the mean fluorescence intensity values were 6.65 ± 3.24 and 16.42 ± 5.78, respectively. Compared to untreated cells, which produced a modest FITC signal (0.16 ± 0.02), this suggested a highly time-dependent cellular uptake compared to the background NLC signal. Hoechst 33342 dye was utilized as a counter-stain (Figure 4).

### 2.5. Pharmacokinetic Parameters

**Figure 5:**
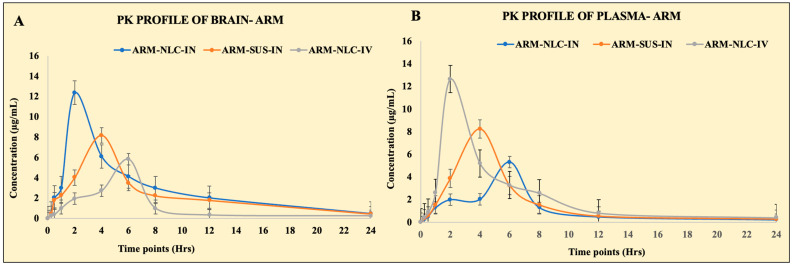
Pharmacokinetic profiles of artemether (ARM) in (**A**) Brain and (**B**) plasma following intravenous (IV) and IN administration of NLCs and free ARM suspension. The results of the investigation, conducted in triplicate (*n* = 3), are presented as the average value plus the standard deviation. The groups were compared through statistical analysis, and a *p*-value of less than 0.005 indicated significance. Significantly, the administration of the NLCs via the IN route demonstrated a high level of significance (*p* < 0.001) when compared to the free ARM suspension group.

IN drug administration enables non-invasive access to the brain through the nose through direct and indirect channels. In the current investigation, we evaluated the levels of ARM in the brain after IN ARM-NLC and ARM suspension administration, and intravenous injection of ARM-NLCs. The concentrations of ARM in the plasma and brain were plotted against time, and pharmacokinetic characteristics such as Cmax, Tmax, AUC, and AUMC, as well as neuro-pharmacokinetic parameters (DTI, DTP, and DTE) were estimated using Equations (2)–(6). To enable comparability, the ARM dose was kept standard. Figure 5 displays the plasma and brain concentrations of ARM produced from the suspension and NLCs after IN and intravenous administration, respectively. Two hours post intravenous administration, the ARM plasma levels were higher than those from the other formulations. Subsequently, two hours post IN administration, the highest plasma concentration of ARM-NLCs was obtained; at four hours, there was no discernible alteration in ARM plasma levels. Fascinatingly, after receiving ARM-NLC injections intravenously and intranasally, ARM plasma concentrations of the mice stayed higher than those that were administered free drug. There was a delay in the increase in plasma concentration for the IN distribution route due to the drug molecules having to pass through the nasal membrane. Contrarily, a higher peak concentration was noted with intravenous infusions since the drug can enter the bloodstream more quickly. There were significant concentration differences between the free medication and the NLCs due to ARM diffusing through the polymer matrix.

Likewise, the brain samples had a greater concentration of ARM at 2 h for intranasally administered ARM-NLCs. Nevertheless, after 4 h, the ARM-NLCs administered intranasally led to a greater concentration of ARM in the brain when compared to the free drug. The brain ARM levels achieved by ARM-NLC were higher than those of other formulations, suggesting enhanced permeation through the nasal mucosa, increased lipophilic nature of the NLC, and superior drug efflux. The pharmacokinetic profile indicates a heightened brain efficiency of ARM when utilized in the NLCs for IN administration. The pharmacokinetic profiles for the blood and brain samples are displayed in Table 1.

### 2.6. Estimation of Targeting Efficiency

In addition, our study thoroughly examined the transportation of ARM to the brain through ARM-loaded NLCs and a free ARM suspension. When ARM-loaded NLCs were administered via the IN route, the DTP and DTE estimates were higher than those of the free ARM suspension. According to the DTP results, the intranasal delivery method allows the medications to bypass the blood–brain barrier and reach the brain. When administered intranasally, the ARM-NLCs showed a DTP value of 0.04%, while the ARM suspension exhibited a DTP value of 0.01%. The brain-targeting effectiveness of the NLCs was further demonstrated by the remarkable DTE value of 502.7% for the NLCs loaded with ARM. Similarly, effective brain delivery was indicated by a DTI of 5.02% (DTI > 1). The higher DTP and DTE values indicate that the drug moieties were transported from the nasal cavity to the brain via the trigeminal and olfactory pathways. Therefore, the improved DTP and DTE estimates in the developed NLCs and the free drug suspension indicate that ARM had a greater capacity to enter the brain through these formulations. In addition, the drug’s encapsulation in the NLCs shields it from degradation and enables it to bypass the efflux mechanisms.

### 2.7. Pharmacodynamic Evaluation: In Vivo Anti-Malarial Efficacy

#### 2.7.1. Parasitaemia Evaluation

**Figure 6:**
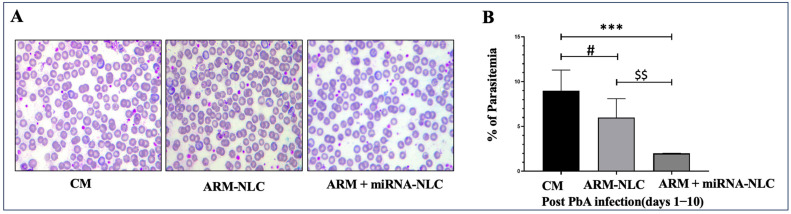
Parasitaemia estimation. (**A**) The photomicrographs show the burden of infected red blood cells (RBCs) and parasites in the untreated/control group and the treated groups (ARM-NLCs and ARM-miRNA-NLCs). (**B**) The parasite burden in mice treated with ARM-NLCs and ARM-miRNA-NLCs was reduced. $$ Demonstrates a significant difference (*p* < 0.01) compared to the treatment groups. *** Denotes a highly significant difference (*p* < 0.001) compared to the CM and ARM+ miRNA-NLCs groups, while # signifies a notable variance (*p* < 0.05) compared to the CM and ARM-NLCs. The data are provided as mean ± SD (*n* = 3). One-way ANOVA was utilized for statistical analysis, followed by Tukey’s multiple comparison test.

The results displayed an evident reduction in parasitaemia in both the ARM-NLC- (*p* < 0.05) and ARM+ miRNA-NLC (*p* < 0.001)-treated groups when contrasted to the CM group. In addition, the group treated with ARM+ miRNA-NLCs exhibited a noteworthy reduction in parasite burden compared to the ARM-NLC-treated group (*p* < 0.01). It appears that the ARM+ miRNA-NLC formulation has exceptional effectiveness in combating malaria (Figure 6). The combination of ARM+ miRNA-NLCs may have prolonged its interaction with parasites, resulting in preserving innate immunity and a reduction in the parasite burden compared to ARM-NLCs.

#### 2.7.2. Histopathology Examinations

In the control group, there was a normal framework in myelinated hippocampal neurons within the hippocampal region and pyramidal neurons in the cerebral cortex, which is consistent with the anticipated baseline conditions. These findings provide a foundation for comprehending the usual histological characteristics without any experimental interventions. In contrast, the CM group showed significant neuropathological alterations, such as severe multifocal degeneration and demyelination of neurons in the cerebral cortex, coupled with foci of inflammation marked by the infiltration of inflammatory cells. Furthermore, the appearance of multifocal necrosis and apoptotic neuron foci in the hippocampus’s dentate gyrus (DG) area emphasized the disease’s adverse impact on brain structures.

**Figure 7:**
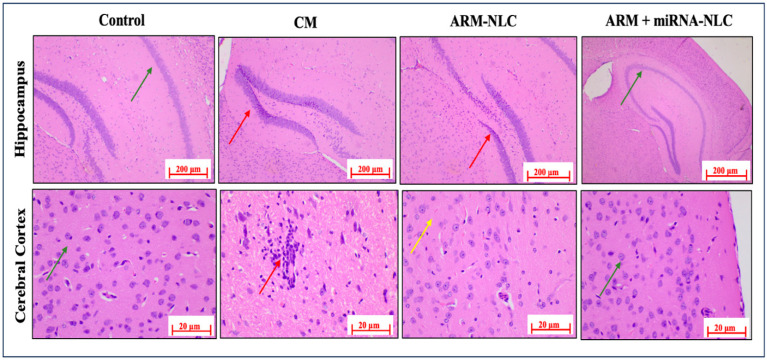
Histopathology H&E staining of hippocampus and cerebral cortex. The control group shows normal myelinated hippocampal neurons (green arrow) and pyramidal neurons of the cerebral cortex (green arrow). The CM disease group shows neuronal degeneration and inflammation in the cerebral cortex (red arrows) and multifocal neuronal necrosis/apoptosis in the hippocampus (red arrow). The ARM-NLC group has moderate inflammation in the cerebral cortex (yellow arrow) and multi-focal neuronal necrosis/apoptosis in the hippocampus (DG and CA4 areas) (red arrow). The ARM+ miRNA-NLC treatment resulted in normal cerebral cortex pyramidal neurons (green arrow) and normal myelinated hippocampus neurons (green arrow).

In the ARM-NLC group, mild multifocal necrosis and apoptotic foci in the DG area of the hippocampus indicated minimal improvement in the observed neuronal degeneration and apoptosis. Conversely, the ARM+ miRNA-NLC group showed a remarkable restorative effect, as evidenced by the restoration of the normal morphology in myelinated hippocampal neurons and pyramidal neurons in the cerebral cortex. These findings imply that the ARM+ miRNA-NLC combination may offer a more optimistic therapeutic strategy, possibly mitigating the neurodegenerative effects observed in the CM and ARM-NLC groups, as shown in Figure 7. Additional investigations are needed to understand the underlying mechanisms better and enhance the combined benefits of this therapeutic approach for treating CM.

#### 2.7.3. Molecular Interplay Driving Pathogenesis in Cerebral Malaria

In the intricate realm of CM, the delicate interaction of molecular factors, particularly the NOX2 gene and IL-6, plays a significant role in shaping the pathogenic processes. Oxidative stress driven by NOX2 contributes to the disruption of the blood–brain barrier, leading to damage to neurons and the development of neurological complications. Concurrently, increased IL-6 levels intensify the inflammatory response, leading to a more pronounced breakdown of the blood–brain barrier and making it easier for immune cells to enter, ultimately causing severe consequences such as seizures and coma [35]. The dysregulation of both NOX2 and IL-6 indicates that they are potential therapeutic targets for reducing the severity of CM and its related neurological consequences.

##### Modulation of Gene Expression

**Figure 8:**
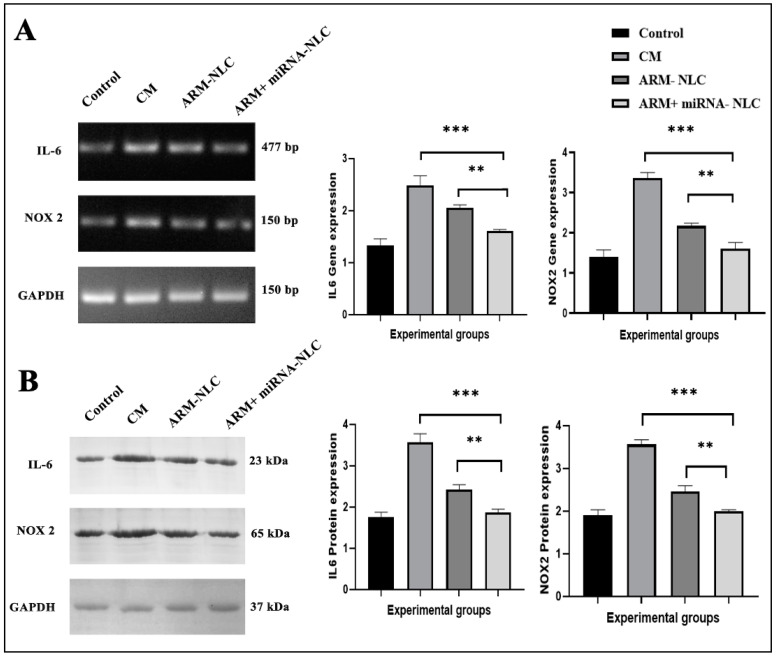
Reduction in the expression of IL-6 and NOX2. (**A**) Expression of the IL-6 and NOX2 in whole brains. The expression levels were normalized using GAPDH, and the densitometric results are shown. (**B**) The Western blot analysis demonstrates the quantification and normalization of the protein expression levels of both the IL-6 and NOX2 genes relative to those of GAPDH. (** denotes *p* < 0.01, *** denotes *p* < 0.001).

The results of the gene expression analysis revealed a noteworthy reduction in IL-6 expression with both the ARM + miRNA-NLC (1.60 ± 0.03, *** *p* < 0.001) and ARM-NLC (2.05 ± 0.06, ** *p* < 0.01) therapies in comparison to the CM group (2.48 ± 0.18) (Figure 8A). The reproducibility of the data was increased by using GAPDH as a loading control, showing a drop in IL-6 as a result of the combination therapy. This reduction was consistent with the theoretical paradigm, implying that focusing on IL-6 might potentially lessen the blood–brain barrier disruption, providing a promising option for therapy. As IL-6 is a key mediator of pro-inflammatory signalling pathways, by targeting IL-6, either through inhibition of its production or blockade of its signalling pathways, it may be possible to reduce the overall inflammatory response in cerebral malaria. This could lead to the decreased activation of endothelial cells and reduced production of inflammatory mediators that contribute to BBB disruption [36]. Moreover, IL-6 is also involved in the activation of astrocytes and microglia, which are resident immune cells in the central nervous system. These cells can produce additional inflammatory mediators that contribute to neuroinflammation and BBB disruption. By reducing IL-6 levels, it may be possible to mitigate the activation of astrocytes and microglia, thereby dampening neuroinflammation and preserving BBB function [37].

Likewise, significant decreases in NOX2 gene expression were seen with the ARM+ miRNA-NLC (1.61 ± 0.15, *** *p* < 0.001) and ARM-NLC (2.17 ± 0.07, ** *p* < 0.01) treatments in comparison to the CM group (3.36 ± 0.14) (Figure 8A). The significant decrease in NOX2 expression highlights the potential of ARM+ miRNA-NLCs and ARM-NLCs in reducing oxidative stress and preventing blood–brain barrier damage, positioning them as highly promising therapeutic approaches in the treatment of CM.

##### Protein Expression Profiling

There was a decrease in IL-6 protein expression with ARM+ miRNA-NLCs (1.86 ± 0.08) compared to the ARM-NLC (2.41 ± 0.12, ** *p* < 0.01) and the CM groups (3.564 ± 0.21, *** *p* < 0.001), which is consistent with the gene expression findings (Figure 8A). The consistent modulation at both the gene and protein levels highlights the substantial effectiveness of ARM+ miRNA-NLCs in reducing IL-6 expression, suggesting a comprehensive strategy to control the inflammatory cascade in CM.

Corresponding to the gene expression findings, the expression of NOX2 protein showed a significant decrease with ARM+ miRNA-NLCs (1.99 ± 0.04) compared to the ARM-NLC (2.46 ± 0.13, ** *p* < 0.01) and the CM groups (3.560 ± 0.11, *** *p* < 0.001) (Figure 8B). The potential of ARM+ miRNA-NLCs to successfully downregulate NOX2 expression is highlighted by the consistent findings at both the transcript and protein levels, suggesting a possible therapeutic pathway for treating the oxidative stress and BBB disruption in CM.

## 3. Discussion

Our research details the formulation of an NLC using the hot homogenization technique for intranasal delivery. Specifically, our focus was on examining the system’s physical characteristics such as particle size, PDI, and zeta potential to enhance brain targeting. The obtained size enables a greater surface area, resulting in optimized ARM release. Due to the monomodal dispersion’s reduced PDI, colloidal dispersion enables the extended release of the drug. The formulation had a higher EE of 93.06 ± 3.43% compared to the earlier results of 91 ± 3.62% published by Vanka Ravisankar et al. [38]. This increased efficiency is due to ARM’s remarkable solubility and strong lipophilic qualities in our formulation, which stimulate drug release at the absorption site. The pharmacokinetic profiles of ARM in the brain illustrate the efficacy of the NLC. After two hours, the brain concentration of the ARM-NLCs administered through the nasal route was higher, surpassing the levels achieved by the ARM suspension. This suggests a higher level of infiltration through the nasal mucosa. The prolonged duration of ARM levels in the brain, lasting up to 4 h, indicates the intriguing potential of NLCs in effectively preventing drug efflux and ensuring a sustained availability of the formulation. The impressive abilities of the ARM-NLCs, surpassing other formulations like the ARM suspension, highlight the lipophilic nature of the developed NLCs and their exceptional ability to cross the blood–brain barrier. In summary, the pharmacokinetic data suggest that including ARM in NLCs for intranasal administration could potentially improve drug bioavailability and facilitate targeted drug delivery to the brain.

The impressive DTE value of 502.7% for the ARM-loaded NLCs provides strong evidence of the brain-targeting efficiency of the NLC. When DTE readings are above 100%, it implies that the delivery to the brain is effective. In addition, the DTI of 5.02% (DTI > 1) provides further evidence of the successful delivery of ARM-NLCs to the brain. The developed NLCs exhibited significantly higher DTP and DTE values compared to the free drug suspension. The results revealed that the ARM-NLCs exhibited enhanced brain permeability, evading the blood–brain barrier and entering the brain through the olfactory bulb. The results highlight the importance of NLC encapsulation in safeguarding against degradation and bypassing efflux mechanisms, enabling direct drug delivery to the brain. The successful encapsulation of miR-223 (10 nM) in NLCs was demonstrated by its ability to inhibit migration under an applied electrical field. The research highlighted the NLCs’ exceptional stability and protective characteristics, which are essential for preserving the therapeutic potential of the loaded miR-223. These findings highlight the strength of ARM-NLCs as a promising vehicle for miRNA delivery.

This study confirmed the successful cellular uptake of FITC-tagged NLCs loaded with ARM-miR-223. Through the time-dependent analysis, it was found that there was significant penetration through the cell membrane. After 6 and 24 h of incubation, the mean fluorescence intensity values were 6.65 ± 3.324 and 16.42 ± 5.78, respectively. The outcomes highlight the promising capabilities of FITC-NLCs in delivering drugs directly into the cellular cytoplasm. The group that received intranasal therapy with ARM+ miRNA-NLCs had a notably lower parasite load than the group that received ARM-NLCs (*p* < 0.001), as shown in Figure 6. This shows that the ARM + miRNA-NLC formulation has better anti-malarial efficacy than ARM-NLCs. The anti-inflammatory, antioxidant, and neuroprotective properties of ARM are well recognized, and miRNAs possess immune-protective characteristics. The study outcomes showed that 10 nm of miRNA and 5 mg/kg of ARM can confer neuroprotection in ECM and eradicate the parasite, protecting the integrity of the brain.

In the histopathology examinations, it was revealed that the control group showed normal histological changes, while the disease group displayed significant neuropathological changes, emphasizing the impact of CM. The ARM-NLC group displayed limited neuronal degeneration, as indicated by the necrosis and apoptosis noticed in the DG region. On the other hand, the ARM+ miRNA-NLC group showed a significant therapeutic effect, indicating a positive therapeutic benefit with the restoration of normal neuronal morphology. These findings indicate the potential of ARM and miRNA-loaded NLCs in reducing neurodegenerative effects, highlighting the importance of additional research to enhance this therapeutic approach for addressing CM. The complex molecular interactions in CM revolve around increased NOX2 and IL-6 signalling which shape the disease’s progression through leveraging ROS and pro-inflammatory cytokine production that causes hippocampal neuronal damage and further intensifies the disruption of the BBB. Interestingly, the intervention was proven to reduce the disease pathologies in ECM. Nevertheless, more investigation is needed to clarify the physiological alterations that helped to preserve the hippocampus in CM. This novel therapeutic approach has the potential to tackle the intricacies of CM, paving the way for future investigations and potential clinical applications in combating this devastating disease.

## 4. Materials and Methods

### 4.1. Chemicals

ARM (purity of <98.8%) was acquired from IPCA Laboratories Ltd. (Mumbai, India), and miRNA-223 was procured from Symbio Technologies (Monmouth Junction, NJ, USA). Precirol and Labrasol were gifts from Gattefosse (Mumbai, Maharashtra, India). The HEK-293 (human embryonic kidney) cell line was sourced from NCCS (Pune, India). DMEM high-glucose media (Cat No: AL111) and Foetal Bovine Serum (#RM10432) were obtained from Himedia, Mumbai, India. Hoechst 33342 dye (Cat No: 15547) and fluorescein isothiocyanate (FITC) dye (Cat No: 46950) were procured from the Cayman Chemical Company (MI, USA) and Sigma-Aldrich (Bangalore, Karnataka, India). *Plasmodium berghei* ANKA was sourced from ICMR-NIMR (New Delhi, India). miR-223′s sense chain sequence is UGUCAGUUUGUCAAAUACCCCA, while its anti-sense chain sequence is GGGUAUUUGACAAACUGACAU.

### 4.2. NLC Formulation

**Figure 9:**
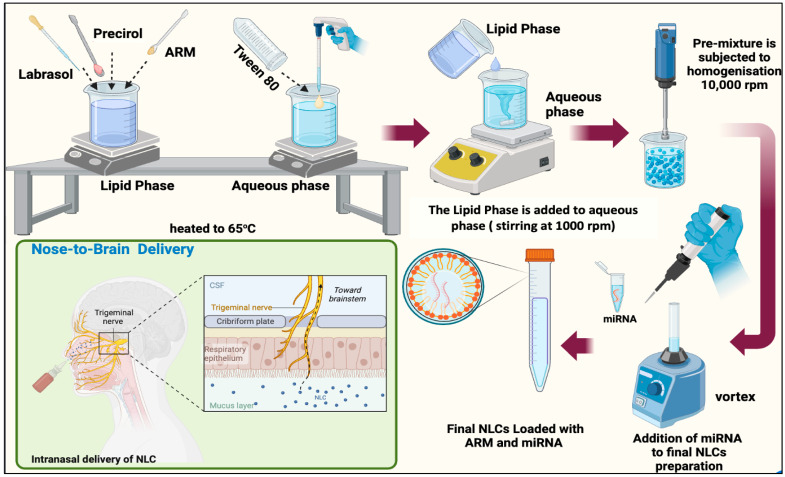
Formulation steps for ARM+ miRNA-NLCs and its potential application for nasal administration.

ARM+ miRNA-NLCs were formulated using the hot-melt homogenization process. Initially, a pre-emulsion was produced by combining the lipid and aqueous phases at 65 °C with the help of a magnetic stirrer (REMI 1 MLH, Mumbai, Maharashtra, India) operating at 1000 rpm [39,40]. The lipid phase consisted of 3% (*w*/*w*) Precirol (solid lipid) and Labrasol 1.5% (*w*/*v*) (liquid lipid), with the addition of 0.1% (*w*/*w*) ARM in its melted state. The aqueous phase contained 0.5% (*w*/*v*) tween-80 (surfactant) in Millipore water. The total mass of the ARM-loaded NLCs was 20 g, and after forming the pre-emulsion, it underwent homogenization at 10,000 rpm using an IKA T25 Ultra Turrax homogenizer (Bangalore, India) for 10 min. Following homogenization, 10 nM of miRNA was embedded into the ARM-NLCs, and the mixture was incubated for 35 min at room temperature (25 °C), as portrayed in Figure 9.

### 4.3. Characterization of ARM+ miRNA-NLCs

#### 4.3.1. Particle Size and Zeta Potential Analyses for Nanostructured Lipid Carrier Systems

The dynamic light scattering approach was employed to measure the mean particle diameter, PDI, and zeta potential (ζ) of the ARM+ miRNA-NLCs employing Nano ZS 90 equipment from Malvern, UK. Before being transferred into a disposable polystyrene cuvette with a 1 cm depth, the samples were diluted 100 times with Milli-Q water (Merck Milli Q Ultrapure Water Purification System, Chennai, Tamil Nadu, India). The average particle diameter and distribution breadth were acquired using this methodology, with the distribution breadth reported as the polydispersity index (PDI). At 25 ± 1 °C, the particle diameter was quantified using light scattering at a 90° angle. The formulation’s zeta potential (ζ) was estimated using transparent disposable zeta cells and Milli-Q water as the dispersion solvent [41].

#### 4.3.2. Encapsulation Efficiency (% EE)

The %EE of the ARM+ miRNA-NLCs was determined using ultrafiltration. This procedure involved centrifuging a millilitre of the NLCs for ten minutes at 4000 rpm in the top chamber of a centrifuge tube with an ultrafilter (Pall Laboratories, 2.5 kDa, Mannheim, Germany) [42]. The quantity of ARM loaded in the NLCs was calculated by subtracting the total amount of ARM incorporated in the formulation of the NLCs from the amount found in the supernatant. The quantity in the filtrate was estimated utilizing RP-HPLC at 209 nm. We calculated the % EE by applying the following equation:

(1) $$\%EE=\frac{Total\;ARM\;used-Free\;ARM\;insupernatant}{Total\;ARM\;used}\times 100$$

### 4.4. miRNA Retardation Assay Using Agarose Gel Electrophoresis

The complexation of miRNA with the ARM-NLCs delivery method was evaluated using agarose gel electrophoresis [43]. The migration of unbound miRNA from the complex was evaluated using an electrophoresis technique, with naked miRNA as a control. Approximately 10 nM of miRNA was combined with NLCs, blank NLCs, ARM-NLCs, ARM+ miRNA-NLCs, ARM+ miRNA-NLCs + heparin, and heparin alone. The reaction took place at 37 °C for one hour. Following the addition of 10 μL of 2X DNA loading dye (containing 0.25% bromophenol, 0.25% xylene cyanol, and 30% glycerol), the samples were placed into a 1.5% agarose gel with 0.5X Tris-acetate-ethylenediaminetetraacetic acid (TAE) buffer and electrophoresed at 5 V/cm for one hour at room temperature. The visualization of nucleic acids involved the incorporation of 0.5 μg/mL ethidium bromide into the agarose gel [44].

### 4.5. Cellular Uptake Visualization through Confocal Laser Scanning Microscopy

Understanding the cellular absorption of nanocarriers is essential for developing effective drug delivery methods. Our current study’s primary aim was to explore and examine the internalization of FITC-dye-tagged NLCs by cells. HEK293 cells were cultured in a 35 mm glass-bottom plate at a density of 0.5 × 105 cells/1 mL for 24 h in a CO2 incubator at 37 °C. After aspirating the spent medium, the cells were exposed to the necessary concentration (5 µL) of NLCs labelled with 10 µg/mL of FITC dye in 1000 µL of culture medium. The cells were incubated in a dark for 6 and 24 h. Post-treatment, the medium was isolated from all wells, which were then washed with phosphate-buffered saline (PBS). Subsequently, the cells were counter-stained with a 5 µg/mL Hoechst 33342 solution. The image analysis was conducted using confocal laser microscopy (Carl Zeiss LSM 880, Leica Microsystems, Mannheim, Germany) and ZEN Blue software of 2.5 version, and the relative fluorescence intensity values of FITC were quantified using Image J software (free version) [45]. This enhanced understanding of the nanoparticle uptake mechanisms contributes to our comprehension of cell interactions with FITC-tagged NLCs and catalyses advances in innovative nanocarriers for drug delivery applications. This advancement holds promise for developing more potent and precisely targeted therapeutic approaches.

### 4.6. RP-HPLC Specifications and Mobile Phase

Drug quantification was performed utilizing RP-HPLC on a Shimadzu I series LC 2030 plus equipment fitted with a UV detector (Shimadzu, Kyoto, Japan). A C18 column (150 × 4.6 mm, 5 µm) was employed, along with a gradient flow pump and an auto-sampler. The mobile phase was a 70:30 *v*/*v* acetonitrile and 10 mM ammonium acetate buffer combination. Elution was carried out at 0.6 mL/min, with an injection volume of 10 µL. The column temperature was held at 35℃, and the detection of the drug peak occurred at 209 nm, with a total runtime of 10 min. The retention times for the internal standard (dihydroartemisinin (DHA)) and ARM were 2.5 and 4.1 min, respectively. The data analysis was executed using Lab Solutions software (Version 5.90) [46].

### 4.7. Pharmacokinetic Parameters and Animal Husbandry

We employed a validated bioanalytical method to perform pharmacokinetic studies on ARM in mice. Female C57BL/6 mice, aged 4–6 weeks (weighing 15–20 g), were procured from Adita biosys Pvt Ltd. (Tumkuru, Karnataka, India). The mice were housed in clean cages under standard laboratory conditions, with access to sterilized bedding, food, and portable mineral water provided ad libitum. The animals went through a seven-day acclimatization period in controlled laboratory conditions. Following an overnight fast with unrestricted access to water, each mouse in the corresponding groups received the treatments given in Table 2. Blood and brain samples were collected at 0, 15, and 30 min, and 1, 2, 4, 6, 8, 12, and 24 h (*n* = 3) post-dosing through a retro-orbital puncture and cervical dislocation. For the sample preparation for HPLC, the protein precipitation method was applied [47].

The collected blood underwent centrifugation for 10 min at 3500 rpm at 4 °C. The extraction of ARM from the plasma involved the addition of 0.3 mL of HPLC-grade acetonitrile. The mixture was then vortexed for 10 min and subjected to a final centrifugation for 10 min at 10,000 rpm to ensure thorough extraction. The ARM concentration in the supernatant was estimated using RP-HPLC. The mice were humanely euthanized through cervical dislocation at specific time intervals after blood collection. The brain samples were obtained by carefully opening the skull, gently rinsing with a saline solution, and cautiously dry blotting them. The brain samples were thoroughly minced in PBS with a pH of 7.4. The brains were extracted and cleaned with PBS to eliminate any clinging tissues and were then homogenized with acetonitrile using a high-speed tissue homogenizer (FastPrep-24TM Classic, MP Biomedicals India Pvt Ltd., Navi Mumbai, India). The homogenate underwent centrifugation at 6000 rpm for 15 min at 4 °C. The resulting supernatant was carefully collected and stored at −20 °C for subsequent analysis. RP-HPLC was used to determine the concentration of ARM in the clear supernatant.

The pharmacokinetic parameters for both the plasma and brain were analysed using concentration–time graphs. Non-compartmental modelling was utilized during the appraisal of the pharmacokinetic metrics for ARM, such as the area under the curve (AUC), time to reach maximum concentration (Tmax), and maximum concentration (Cmax). These specifications can reveal valuable insights into the drug’s activity in the body and its clinical applications. The experiments were conducted with precision, revealing critical information on ARM’s pharmacokinetics. The pharmacokinetic metrics for ARM were subjected to statistical evaluation using GraphPad Prism version 9.0 software (GraphPad Software, San Diego, CA, USA), utilizing paired *t*-tests to examine the probability value.

### 4.8. Assessment of Targeting Efficiency

The partition coefficient (Kp) of the brain-to-plasma concentration ratio can be applied to compare the targeting efficacy of ARM-NLCs and the ARM suspension following IN administration. This coefficient is derived as the proportion of Cbrain (brain concentration) to Cplasma (plasma concentration). A high Kp following IN treatment reflects strong brain targeting. The metrics adopted for estimating the targeting efficiency include the drug targeting index (DTI), direct transport percentage (DTP), and drug targeting efficiency (DTE) [48,49].

(2) % DTE=AUC0−24brainINAUC0−24bloodINAUC0−24brainIVAUC0−24bloodIV×100

(3) $$\%\;\mathrm{DTP}=\frac{AUC0-24\left(brain\right)IN-(\frac{AUC0-24\left(brain\right)IV}{AUC0-24\left(blood\right)IV}\times AUC0-24(blood)IN)}{AUC0-24(brain)IN}\times 100$$

(4) $$\mathrm{DTI}=\frac{\left[AUC\right.(brain)/AUC\left.\left(Blood\right)\right]IN}{\left[AUC\right.(brain)/AUC\left.\left(Blood\right)\right]IV}$$

(5) $$\mathrm{Absolute}\;\mathrm{bioavailability}=\frac{AUC(brain)IN(NLCsFormulation)}{AUC(brain)IV(NLCsFormulation)}\times 100$$

(6) $$\mathrm{Relative}\;\mathrm{bioavailability}=\frac{AUC(brain)IN(NLCsFormulation)}{AUC(brain)IN(DrugSuspension)}\times 100$$

### 4.9. In Vivo Assessment of Antimalarial Effectiveness in Mice Infected with Plasmodium berghei ANKA (PbA)

*Plasmodium berghei ANKA* (PbA) of parasitic blood stages (1 × 10^6^) was diluted in 200 µL of sterile 1x PBS at pH 7.4 was intraperitoneally injected into 24 female mice. The choice to focus on this distinct age group was made due to the observation that elderly mice with PbA infection frequently lack obvious signs of CM [50]. The investigation concentrated on animals that exhibited PbA infection. The mice had unlimited access to conventional food and water during the study and the treatments are listed in Table 3. Ketamine (150 mg/kg) and xylazine (10 mg/kg) (Raghu Lal Chemicals, Mysuru, Karnataka, India) were used to as anaesthetics for essential operations.

#### 4.9.1. Evaluation of Parasitaemia in ECM

Parasitaemia was determined in each of the groups by examining Giemsa-stained blood smears obtained from the caudal vein under a microscope. The obtained smears were stained for 20 min at ambient temperature and examined using an Olympus BX51 (Life Science Technology, Tokyo, Japan) light microscope at a 100× resolution. We closely observed the mice which were infected with the parasite until they displayed symptoms of CM. Afterwards, they underwent a one-week treatment with ARM-NLCs and ARM+ miRNA-NLCs to prevent the recurrence of parasites. The treatment began on the 6th day post infection, coinciding with the appearance of parasitaemia.

The parasitaemia percentage was computed as follows:

(7) $$\%Parasitaemia=\frac{Number\;of\;parasite\;infected\;red\;blood\;cells\;(RBCs)}{Total\;no\;of\;RBCs}\times 100$$

#### 4.9.2. Histopathological Analysis: Brain Examination through Haematoxylin and Eosin (H&E) Staining

Following euthanasia, the animals were subjected to intracardial perfusion using a saline solution, followed by a chilled solution containing 4% paraformaldehyde (PFA). All the brain specimens were subsequently gathered and preserved in a 4% PFA solution. H&E staining, a broadly used method for analysing tissue changes in different diseases, was conducted on 10 μm thick sections of the hippocampus. The Harris haematoxylin stain was applied to all brain sections using well-established techniques. Images of the stained sections were obtained with an Olympus BX-51 microscope at 100× and 40× magnification [51,52].

#### 4.9.3. Evaluation of Brain Samples through Reverse Transcription Quantitative PCR (RT-qPCR) and Western Blot Analysis

Total RNA was derived from whole brain tissues in each experimental group (n = 4 per group) using Trizol reagent (T9424 Sigma Aldrich, Mumbai, India). A NanoDropTM 2000 UV–visible spectrophotometer (Shimadzu, Bangalore, India) was used to quantify the RNA. Following that, cDNA was synthesized from 1 μg of RNA using the Takara Bio PrimeScriptTM 1st Strand cDNA Synthesis kit (6110A Takara Biotechnology, Dalian, China). For the PCR process, 5 μL of 2x Dreamtaq Green PCR master mix (K1081 Thermo Fisher Scientific, Pittsburgh, PA) was combined with 0.5 μg/L of cDNA (0.5 μL) and 10 pM of both the forward and reverse primers (1 μL each). Water devoid of nuclease (3.5 μL) was added to bring the total reaction volume to 10 μL. Semi-quantitative PCR was performed with the mixture for 25 cycles using an Applied Biosystems Veriti 96-well Thermal Cycler (Applied Biosystems, Foster city, CA, USA). The PCR technique included a 30 s denaturation phase at 95 °C (stage 1) and a 2 min denaturation step at 95 °C (stage 2). The annealing stage took 45 s at the proper melting temperature (Tm), while the extension step took 5 min at 72 °C [53,54]. Integrated DNA Technologies (IDT) provided the primers for the IL-6, NOX2, and GAPDH genes, and their sequences are shown in Table 4.

#### 4.9.4. Western Blot

Before homogenization, whole brain samples were lysed with sucrose Radio-Immunoprecipitation Assay buffer (RIPA), which included 0.32 M sucrose, 10 mM Tris-HCl, 0.5% sodium deoxycholate, 150 mM NaCl, and 1% NP-40 and was adjusted to pH 7.2. A Dounce homogenizer was employed for blending the mixture at 4 °C, and 10 μL of protease inhibitor (P0044, Sigma-Aldrich, Mumbai, India) was added for every 1 mL of solution. The samples were subjected to the Bradford technique for protein quantification, and 50 μg of proteins was extracted on a 10% SDS-PAGE (Sodium Dodecyl Sulphate-Poly Acrylamide Gel Electrophoresis) gel. The proteins were transferred onto a nitrocellulose membrane immersed in Towbin buffer (Tris-HCl, 3 g; glycine, 14.4 g; methanol, 200 mL; deionized water, 800 mL; pH 8.3) and left to incubate for several hours at 4 °C. The membranes were then blocked for 1 h at room temperature in a buffer containing 5% skimmed milk. The membrane was then incubated at 4 °C for an entire night with primary antibodies, specifically, GAPDH (#5174, Cell Signalling Technology, Danvers, MA USA) and NOX2 rabbit-raised polyclonal antibodies (Abcam, ab80508) at a dilution of 1:1000. This was followed by a 5 min wash with a Tris-buffered PBS solution containing 0.05% Tween 20 (TBST). Following the TBST wash, an alkaline phosphatase-conjugated secondary antibody for rabbit IgG (1:30,000) (whole molecule) (A3687, Sigma-Aldrich, Mumbai, India) was employed to probe the membrane for two hours at room temperature. The immunoreactivity was detected by adding 120 μL solution of BCIP (5-bromo-4-chloro-3-indolyl-phosphate) and NBT (Nitro Blue Tetrazolium, Sigma-Aldrich, Mumbai, India) to an alkaline phosphatase buffer. After applying this mixture to the membrane, it was left in a dark area at room temperature for 5–10 min [55,56].

## 5. Conclusions

To summarize, our study focused on creating highly efficient NLCs for effectively delivering ARM to the brain through the nasal route. The NLCs displayed impressive attributes, with a particle size of 94.39 nm and a remarkable entrapment efficiency of 93.06%. The pharmacokinetic profiles exhibited enhanced drug bioavailability, with higher concentrations in the brain after administration of ARM-NLCs intranasally. The encapsulation of miR-223 in NLCs and the noteworthy cellular uptake demonstrated the stability and potential of the carrier for effective drug delivery.

In the context of CM, the ARM + miRNA-NLC treatment demonstrated remarkable anti-malarial effectiveness, leading to a notable decline in parasite burden that surpassed the results of the ARM-NLC treatment alone. Finally, the histological investigations illustrated the deleterious consequences of CM on brain structures and studied possible interventions. While the ARM-NLC group displayed limited effects, the ARM+ miRNA-NLC group showcased a promising beneficial effect, indicating that the use of ARM+ miRNA-NLCs is a potential therapeutic approach. Interestingly, the molecular effects of CM, especially the effects on NOX2 and IL-6 levels, were predominantly reduced by the therapy compared to the CM group. Additional research is needed to optimize the benefits of ARM and miRNA-NLCs for the efficient management of CM.

Our research highlights the strength of NLCs as a carrier system for IN drug delivery. They provided improved drug bioavailability, effective brain targeting, and feasible applications in treating CM.

## Figures and Tables

**Table 1:** Estimation of pharmacokinetic parameters for ARM-NLCs in brain and plasma.

Pharmacokinetic Parameter	Route of Administration and Nature of Formulation					
	NLCs Loaded with ARM (IN)		ARM Suspension (IN)		NLCs Loaded with ARM (IV)	
	Brain	Plasma	Brain	Plasma	Brain	Plasma
C_max_ (µg/mL)	12.36 ± 0.18	5.30 ± 0.51	8.16 ± 1.01	8.24 ± 0.96	5.82 ± 0.45	12.64 ± 0.95
T_max_ (h)	2	6	4	4	6	2
AUC_0–24h_ (µg·h/mL)	72.88 ± 5.50	27.32 ± 3.79	54.38 ± 4.59	40.08 ± 9.45	28.13 ± 8.16	58.45 ± 7.55
AUC_0–∞_ (µg·h/mL)	78.23 ± 6.72	29.08 ± 6.59	58.96 ± 8.99	42.62 ± 8.57	30.60 ± 11.35	62.32 ± 11.45
AUMC_0–24_ (µg·h^2^/mL)	488.12 ± 26.54	177.92 ± 16.46	403.04 ± 10.88	226.36 ± 23.01	176.76 ± 11.62	323.92 ± 12.77
AUMC_0–∞_ (µg·h^2^/mL)	1054.85 ± 21.56	348.27 ± 16.46	906.30 ± 19.76	647.93 ± 23.01	393.50 ± 18.55	652.81 ± 12.77
K_el_ (h^−1^)	0.10 ± 0.08	0.09 ± 0.06	0.089 ± 0.06	0.10 ± 0.09	0.086 ± 0.12	0.11 ± 0.13
T1/2	8.01	5.98	7.73	6.45	6.69	7.12
MRT_0–∞_	17.88	10.47	12.85	11.97	11.58	15.19
Relative bioavailability	132.95 ± 10.56					
Absolute bioavailability	256.77 ± 14.80					

The findings are expressed as mean ± SD; *n* = 3. The comparisons were considered significant if *p* < 0.05. The results for the NLC formulation through the IN route were significantly different (*p* < 0.001) from the free ARM drug suspension.

**Table 2:** Treatments for pharmacokinetic studies.

Group Name	Administration Route	Description	Dosage
ARM suspension (free drug)	IN	ARM dispersed freely in PBS solution was administered through IN route to the mice.	5 mg/kg
ARM-NLCs	IN	Mice received IN administration of ARM-loaded NLCs.	5 mg/kg
ARM-NLCs	Intravenous (IV)	Mice were administered ARM-loaded NLCs intravenously.	5 mg/kg

**Table 3:** Treatments for pharmacodynamic studies.

Group and Substance	No. of Animals	Description	Treatment
Control	6	No malaria infection	No treatment
CM	8	Female mice infected intraperitoneally with 1 × 10^6^ *Plasmodium berghei ANKA* (PbA) of parasitic blood stages diluted in 200 µL chilled sterile 1x PBS pH 7.4.	No treatment
ARM-NLCs	8	Animals with PbA infection exhibiting neurological symptoms were considered for the experiment.	ARM-NLCs were administered intranasally at a dosage of 5 mg/kg per day for a period of 7 days in a 40 µL volume.
ARM+ miRNA-NLCs	8	The infected mice showing behavioural symptoms were chosen.	ARM-miRNA-NLCs were administered intranasally at a dosage of 5 mg/kg of the drug and 10 nmol of the miRNA daily for a duration of 7 days.

**Table 4:** Primers used for RT-qPCR.

	NCBI Reference Sequence	Sequence (5′->3′)	Length	Product Length	Annealing Temperature (°C)
IL-6					
Forward	Mus musculus IL6 Cybb X54542.1	TTGCCTTCTTGGGACTGATGC	21	187	55.8
Reverse		TTGGAAATTGGGGTAGGAAGGA	22		
NOX2					
Forward	Mus musculus Nox2 (Cybb) Fj168469.1	TGGAAACCCTCCTATGACTTG	24	216	57.5
Reverse		AACTTGGATACCTTGGGGCAC	24		
GAPDH					
Forward	NM_008084.3	GTGTGAACGGATTTGGCCGTATTG	24	146	58.8
Reverse		TTTGCCGTGAGTGGAGTCATACTG	24		

## Data Availability

Data are contained within the article and Supplementary Materials.

## Supplementary Materials

The following supporting information can be downloaded at: https://www.mdpi.com/article/10.3390/ph17040466/s1. Figure S1: Overlain FT-IR spectra of (A) FITC, (B) ARM-miRNA-NLCs, and (C) FITC-ARM-miRNA-NLCs.
