# Supplementary material for: Pharmacokinetics and Pharmacodynamics of a Nanostructured Lipid Carrier Co-Encapsulating Artemether and miRNA for Mitigating Cerebral Malaria
Source: Pharmaceuticals (Basel). 2024 Apr 6;17(4):466. doi: 10.3390/ph17040466 (PMC11053970; doi:10.3390/ph17040466)
Supplement: Supplementary file 1 [file pharmaceuticals-17-00466-s001.zip › pharmaceuticals-2919843-supplementary.pdf]

*pharmaceuticals*

# *Supplementary Data*

*Article*

# Pharmacokinetics and Pharmacodynamics of a Nanostructured Lipid Carrier Co-Encapsulating Artemether and miRNA for Mitigating Cerebral Malaria

**Supplementary Data**

**FT-IR Spectrophotometric Analysis**

For confirming the chemical conjugation between FITC and the lipids of NLCs, FT-IR spectrophotometric analysis was done and recorded overlain FT-IR spectra of FITC, ARM-miRNA-NLCs, and FITC-ARM-miRNA-NLCs are depicted in supplementary Figure S1. The recorded FT-IR spectrum of FITC exhibited a hydroxy characteristic peak at 3423 $cm^{-1}$ and a strong isothiocyanate characteristic peak at 2015 $cm^{-1}$ (Figure S1A). Also, a characteristic peak assigned to the carbonyl group of lactone, at around 1727 $cm^{-1}$ was noted. Furthermore, the absorption peaks at 1594 $cm^{-1}$, 1535 $cm^{-1}$, and 1458 $cm^{-1}$ were characteristic peaks of benzene ring stretching vibration.

On the other hand, in Figure S1B and S1C the characteristic peaks of lipid at 1740 $cm^{-1}$ corroborating to the stretching vibration in the ester bonds was experiential. Furthermore, the peak at 3330 $cm^{-1}$ to 3500 $cm^{-1}$ was attributed to the stretching vibration of methyl groups. In comparison with FITC spectrum, the isothiocyanate characteristic peak at 2015 $cm^{-1}$ disappeared in the FT-IR spectrum of FITC-ARM-miRNA-NLC; which suggested and confirmed that the isothiocyanate group was involved in the addition reaction with the hydroxyl group present on the NLCs (Figure S1C). These outcomes confirmed that FITC dye was conjugated to the NLCs through a chemical conjugation process forming a covalent bond rather than encapsulation.

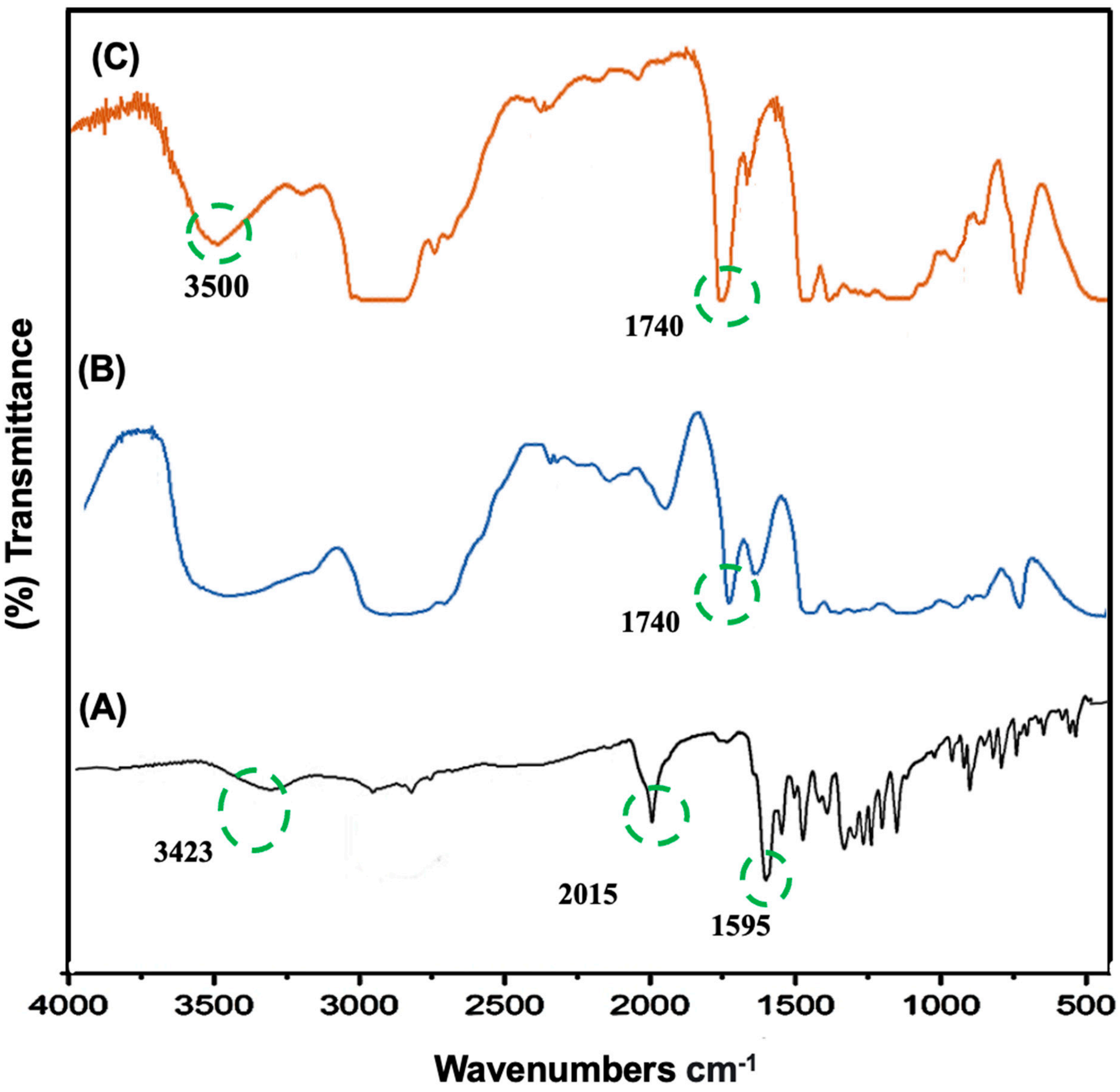


**Supplementary Figure S1:** Overlain FT-IR spectra of (A) FITC, (B) ARM-miRNA-NLCs, and (C) FITC-ARM-miRNA-NLCs.
